# Genome-wide and SNP network analyses reveal genetic control of spikelet sterility and yield-related traits in wheat

**DOI:** 10.1038/s41598-020-59004-4

**Published:** 2020-02-07

**Authors:** Ahmad M. Alqudah, Jemanesh K. Haile, Dalia Z. Alomari, Curtis J. Pozniak, Borislav Kobiljski, Andreas Börner

**Affiliations:** 1 Research Group Resources Genetics and Reproduction, Department Genebank, Leibniz Institute of Plant Genetics and Crop Plant Research (IPK) Corrensstr. 3, OT Gatersleben, D-06466 Stadt, Seeland Germany; 2 Department of Plant Sciences, Crop Development Centre, University of Saskatchewan, Saskatoon, Canada; 3 Research Group Gene and Genome Mapping, Department of Breeding Research, Leibniz Institute of Plant Genetics and Crop Plant Research (IPK) Corrensstr. 3, OT Gatersleben, D-06466 Stadt, Seeland Germany; 4 Biogranum, R&D in agriculture, Toplice Milana 20, Novi Sad, 21000 Serbia

**Keywords:** Natural variation in plants, Plant breeding, Plant genetics

## Abstract

Revealing the genetic factors underlying yield and agronomic traits in wheat are an imperative need for covering the global food demand. Yield boosting requires a deep understanding of the genetic basis of grain yield-related traits (e.g., spikelet fertility and sterility). Here, we have detected much natural variation among ancient hexaploid wheat accessions in twenty-two agronomic traits collected over eight years of field experiments. A genome-wide association study (GWAS) using 15 K single nucleotide polymorphisms (SNPs) was applied to detect the genetic basis of studied traits. Subsequently, the GWAS output was reinforced via other statistical and bioinformatics analyses to detect putative candidate genes. Applying the genome-wide SNP-phenotype network defined the most decisive SNPs underlying the traits. Six pivotal SNPs, co-located physically within the genes encoding enzymes, hormone response, metal ion transport, and response to oxidative stress have been identified. Of these, metal ion transport and *Gibberellin* 2*-oxidases* (*GA*2*oxs*) genes showed strong involvement in controlling the spikelet sterility, which had not been reported previously in wheat. SNP-gene haplotype analysis confirmed that these SNPs influence spikelet sterility, especially the SNP co-located on the exon of the *GA2ox* gene. Interestingly, these genes were highly expressed in the grain and spike, demonstrating their pivotal role in controlling the trait. The integrative analysis strategy applied in this study, including GWAS, SNP-phenotype network, SNP-gene haplotype, expression analysis, and genome-wide prediction (GP), empower the identification of functional SNPs and causal genes. GP outputs obtained in this study are encouraging for the implementation of the traits to accelerate yield improvement by making an early prediction of complex yield-related traits in wheat. Our findings demonstrate the usefulness of the ancient wheat material as a valuable resource for yield-boosting. This is the first comprehensive genome-wide analysis for spikelet sterility in wheat, and the results provide insights into yield improvement.

## Introduction

Bread wheat (*Triticum aestivum* L.) is the second most important cereal crop that has a direct impact on daily food consumption for much of the world population^1^. Current climate change scenarios predict more abiotic and biotic stress events in the areas of wheat production (e.g., Europe)^2^. It is, therefore, necessary to develop cultivars with high yields and better adaptation to stress conditions, in order to ensure food security and social ease^3^ by fine-tuning the genetically complex yield traits. This needs a better understanding of the genetic basis of such traits.

Improving yield-related traits is one of the main aims of wheat breeding programs since inception. Improvement in such complex quantitative traits is difficult because it is a polygenic trait controlled by several genes and are influenced by environmental conditions (e.g., flowering time, (FL)). Wheat grain yield improvement is mainly determined through a combination of many traits controlling final grain yield number e.g. spikes number per plant or area and grain weight. Wheat grain yield was successfully increased by introducing *Rht* genes that in turn increased assimilate partitioning efficiency, and then improving grain yield traits e.g. grain weight^4^. There is clear evidence that most agronomic traits are inherited and controlled by many quantitative trait loci (QTL), for instance, total number of spikelets per spike (TSS)^5–7^, grain number per fertile spikelet (GNFS)^5,6,8^ and thousand kernel weight (TKW)^6,9–11^. Accordingly, promising approaches to exploit the genetic variation and gene identification of these traits is the target for improving yield.

Recently published wheat genome sequences with the high-quality annotated reference genome^12^ make genome-wide analyses more beneficial that empowers researchers for deep genetic analysis, of complex traits in wheat. It is also helps breeders incorporate the discovered causative allele(s) efficiently in breeding programs, to improve yield and adaptation to specific regional conditions. Such progress in wheat production and acclimation is an imperative need to meet the demands of human population growth.

Genome-wide analyses considering multi-traits, multi-years/environments, and multi-loci, along with the high-density SNP (single-nucleotide polymorphism) array are promising to boost power and accuracy in identifying aggregate effects of the locus/gene, and complement plant breeding programs^13^. Genome-wide association study (GWAS) is a powerful tool for dissecting complex traits, by finding causative allelic variation at individual SNP markers or loci (multi-SNPs within linkage disequilibrium [LD] range) that are associated with natural phenotypic variation^14^. In contrast to GWAS, genome-wide prediction (GP) incorporates whole genotypic information from all available marker sequences to predicate the genomic estimated breeding values (GEBVs). Fitting all markers simultaneously avoids multiple testing and the need to identify markers-trait associations based on an arbitrarily chosen significance threshold. In wheat, many reports have used such advanced genome-wide analyses with high-density SNPs e.g. GWAS to understand the natural variation of yield-related traits^15^, and e.g. GP to show that these traits, which were evaluated under multi-environmental conditions, can be predicted^16,17^. GWAS revealed shared QTL between assimilate partitioning efficiency, floret fertility, yield potential, and spike morphology under controlled conditions (greenhouse), suggesting a potential genetic association that controls these complex traits^8^. Allelic variation at *Rht-D1* and *Ppd-D1* has an impact on the natural variation of grain number per spikelet, spikelet fertility, and TKW under field conditions^6^.

In the current study, we analyzed the natural phenotypic and genetic variation of 22 agronomic and spikelet fertility-related traits in one of the oldest association collections in wheat^18,7^, which were phenotyped under field conditions for several years, and genotyped with high-density gene-based SNP (15 K Infinium SNP) arrays. To achieve our objectives, GWAS was carried out to identify the natural genotypic variation and genetic basis of the traits. Use the recent published wheat physical map^12^, SNP arrays provided an unprecedented resolution to identify candidate genes underlying the studied traits^19^. The most important SNPs controlling the studied traits had been detected by a genotype-phenotype network^20^ as the first report in cereals including wheat. Moreover, we tested the potential of GP on yield-related traits, as it enhances the genetic gain of quantitative traits, by accelerating the breeding cycle and increasing selection intensity, which in turn contributes to yield improvement. Interestingly, newly identified candidate genes controlling spikelet sterility, which is annotated as hormone response, metal ion transport, and response to oxidative stress, have been identified. The function of candidate genes was further validated by SNP-gene haplotype and expression analyses.

## Results

### Phenotypic variation and diversity

**Figure 1:**
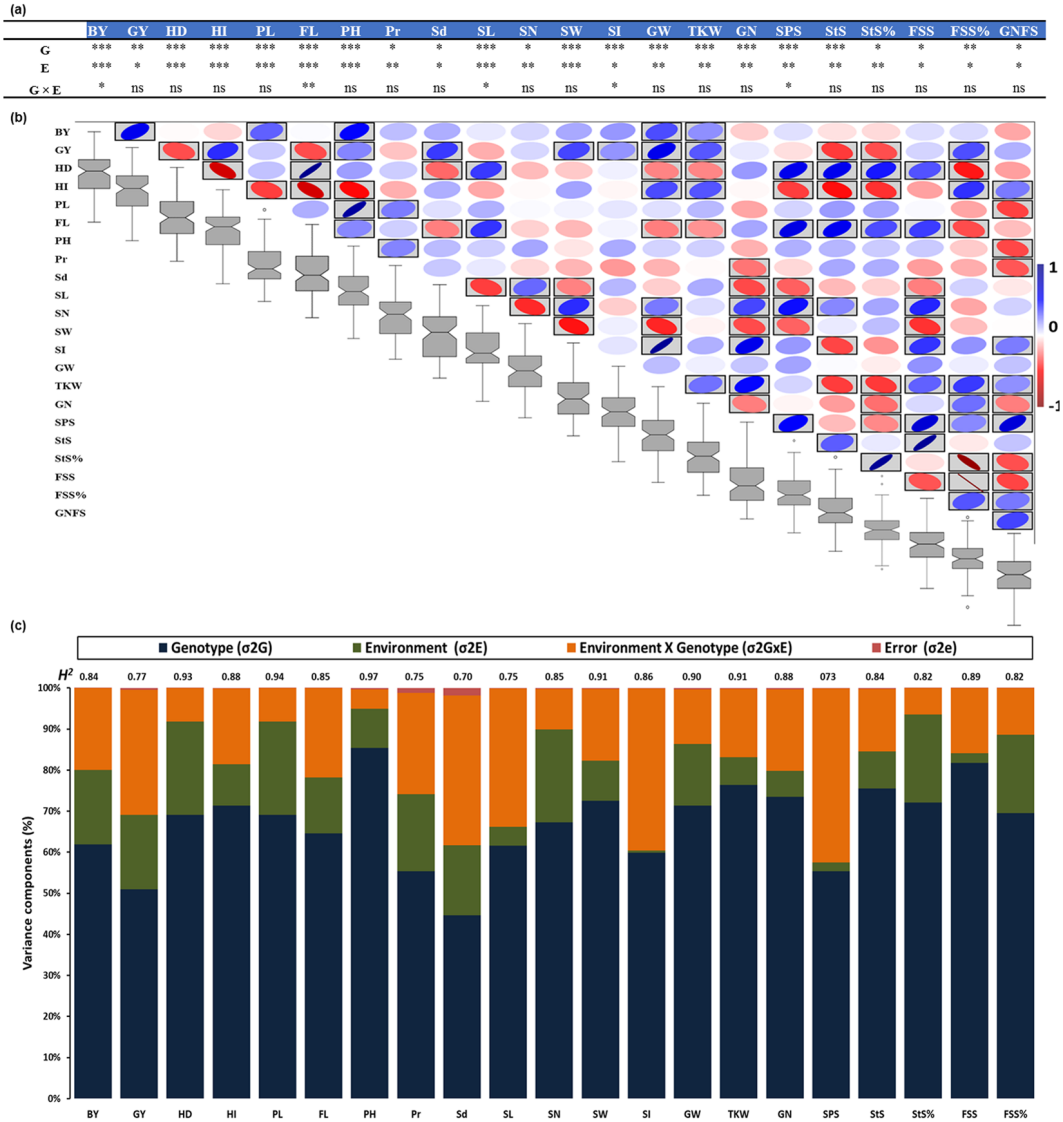
Analysis of variance (ANOVA) of the studied traits (**a**). The correlation matrix among the traits and boxplots (**b**). Variance component analysis and broad-sense heritability (**c**). The degree of significance indicated as *P, 0.05; **P, 0.01; ***P, 0.001; ns: not significant. Where: BY - biological yield, GY – grain yield, HD – heading date, HI – harvest index, PL – peduncle length, FL – flowering time, PH – plant height, Pr – protein content, Sd - sedimentation value, SL – spike length, SN - spike number per m^2^, SW – spike weight, SI – spike index, GW – grain weight per spike, TKW - thousand kernel weight, GN - grain number per spike, SPS - spikelets per spike, StS – sterile spikelets per spike, FSS - fertile spikelet per spike and GNFS - grain number per fertile spikelets.

Significant natural phenotypic variation (at P < 0.05) was identified among the accessions for all of the 22 agronomic traits across the environments based on best linear unbiased estimato (BLUE) values (Fig. 1a). The significant phenotypic variation in the studied traits was also detected among environments at P < 0.05, while G × E was not significant for most of the traits (Fig. 1a). The natural phenotypic variation in the traits was also confirmed by the values of the range, standard error, variation, standard deviation, and coefficient of variation (Fig. 1a).

The Pearson’s correlation coefficients, based on BLUEs for the 22 agronomic traits, ranged from −0.95 to 0.95 (Fig. 1b and Supplementary Table 1). Grain yield (GY), heading date (HD), FL, harvest index (HI), spike number per m^2^ (SN), and spike length (SL) were significantly correlated with most of the agronomic traits. GY had a positive significant correlation with yield-related traits (e.g., grain weight per spike (GW) and TKW), but was negatively correlated with FL, HD, and sterile spikelets per spike (StS). HD and FL are tightly correlated with each other, and positively correlated with SL, spikelets per spike (SPS), and StS, whereas both are negatively correlated with HI and fertile spikelet per spike (FSS). A significant negative correlation at P < 0.05 was found between HI with peduncle length (PL), plant height (PH), SPS, and StS, whereas HI had a significant positive correlation with GW, TKW, and FSS%. SN was also significantly and negatively correlated at P < 0.05 with most of the grain yield-related traits (e.g., spike weight (SW), grain number per spike (GN), GW and SPS; Supplementary Table 1). Additionally, SL was positively correlated with SW, GW, GN, SPS, and FSS.

Broad-sense heritabilities (*H*^2^) ranging from 0.70 to 0.97 were obtained for each trait (Fig. 1c). Most of the agronomic traits (developmental and morphological traits) exhibited relatively high heritability values (≥0.80), whereas only five grain and spike morphological and yield-related traits (GY, SL, protein content (Pr), sedimentation value (Sd) and SPS) had high heritability (0.70–0.77; Fig. 1c). These findings indicate that the population had been selected in a proper way for having high phenotypic variation among genotypes with a low influence of environmental factors on the studied traits.

### Molecular markers, population structure and linkage disequilibrium (LD)

The core collection of wheat accessions belongs to seven geographical regions. In total, 13 K polymorphic SNPs were obtained for the investigated accessions, and 11,220 SNPs passed the filtration criteria (i.e. missing values of ≤10%, and minor allele frequency [MAF] of ≥5%). Of these markers, 10,653 SNPs were genetically mapped and anchored by physical positions according to International Wheat Genome Sequencing consortium (IWGSC), *et al*.^12^, which were then used for analysis. The total map length was 3,511 centimorgans (cM) or ~15 GbP (Giga base pairs), with an average marker density of three SNPs per cM. Information on the number of markers for each chromosome, with the map length and marker density for each chromosome, are presented in Supplementary Table 2. Chromosome 2B carried the largest number of markers (977 SNPs), while the chromosome 6B showed the highest marker density (6.4 markers per cM or one SNP per 4.3 Mbp). Chromosome 4D contained only 50 markers, which was the lowest marker density among chromosomes.

**Figure 2:**
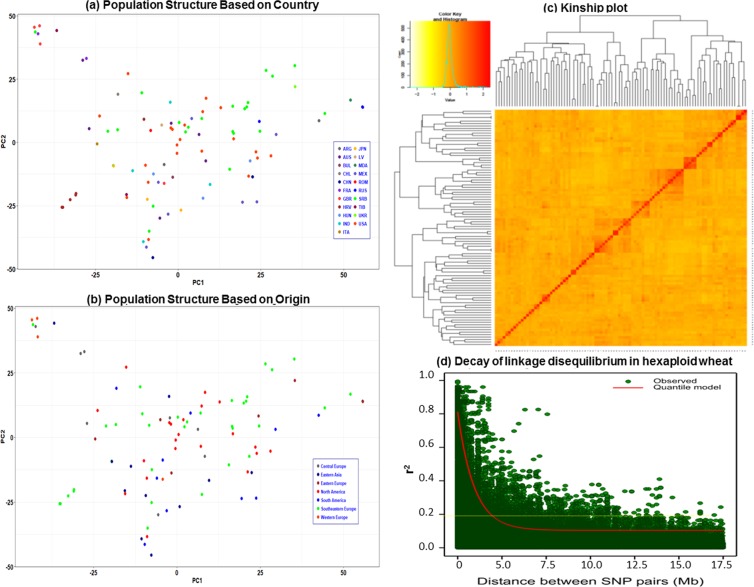
The population structure based on the country (**a**), and region of origin (**b**). Kinship plot showing the relationship among the genotypes (**c**). The decay of linkage disequilibrium in the hexaploid wheat population (**d**).

There are no clear clusters among the accessions with their country and/or origin of the region in the collection, based on the genetic clusters revealed by PCA, using the polymorphic SNP (Fig. 2a,b). Heatmaps and dendrograms of the kinship matrix estimated using GAPIT, based on the polymorphic markers for the accessions, confirmed that there are no clear clusters among the accessions (Fig. 2c). The mean r^2^ values for the whole hexaploid wheat genome gradually decreased with increasing distance between SNPs (Fig. 2d). The average LD decay distance for the whole genome was approximately 5 Mbp (Fig. 2d).

### Significant loci associated with agronomic traits

**Figure 3:**
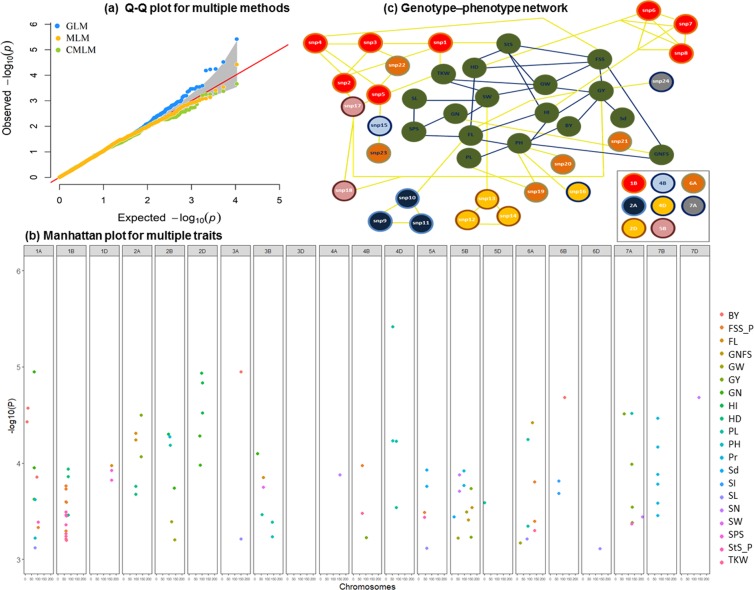
Quantile-Quantile (Q-Q) plot for multiple statistical models (**a**). Manhattan plot for multiple traits with the associated SNPs which have −log10 ≥ 3 (**b**). Genotype-phenotype network analysis (**c**). Where: BY - biological yield, GY – grain yield, HD – heading date, HI – harvest index, PL – peduncle length, FL – flowering time, PH – plant height, Sd - sedimentation value, SL – spike length, SW – spike weight, GW – grain weight per spike, TKW - thousand kernel weight, GN - grain number per spike, SPS - spikelets per spike, StS – sterile spikelets per spike, FSS - fertile spikelet per spike and GNFS - grain number per fertile spikelets.

The comparison among different statistical models showed that the mixed linear model (MLM) model had a strong power to control false positive associations with informative output in all studied traits, compared with general-linear model (GLM), and compressed MLM (CMLM). MLM, which specifies the number of PCs and kinship matrix as a correction for population structure leads to avoiding false positive associations, had been used. The strength of the models had been visualized by a cumulative Quantile-Quantile (QQ) plot of expected vs. observed p-value (Fig. 3a). The false discovery rate (FDR) threshold (−log_10_(P) ≥ FDR) was used to identify significant marker-trait associations (MTAs). Out of 10,653 SNPs used in the GWAS, 118 SNPs had significant −log_10_ (p-value) ≥ passing FDR at 0.01 (Fig. 3b and Supplementary Table 3).

The highest number of associated SNPs for the 22 studied traits were located on chromosomes 1B (17 SNPs), 3B (12 SNPs), 1A and 5B (11 SNPs each), and less than 10 SNPs for the rest of chromosomes (Fig. 3b and Supplementary Table 3). Of these significant SNP markers, the highest number of associated markers was detected for PL (13 SNPs), followed by FSS (10 SNPs) and GY, Pr, and StS (9 SNPs each). Seven SNPs were found to be associated with GN, whereas six SNPs were associated with TKW and HD (Fig. 3b, Supplementary Figs. 1 and 2). Only five SNPs were associated with SL, SN, SW, FL and BY, whereas less than five SNPs were associated with each of the remaining traits. The associated genomic regions were classified as trait-specific or multi-trait (e.g., 1A; 58704752–94120856 bp) was associated with GN by 3 SNPs, whereas 1B (142834452–278473822 bp) was associated with FSS, StS, and TKW by 14 SNPs (Supplementary Figs. 1 and 2). Twelve SNPs with −log_10_(≥4.5) were distributed over 8 chromosomes, and mostly associated with grain yield-related traits (Fig. 3b and Supplementary Table 3).

The highest −log_10_ (5.4) was detected for the marker (*Kukri_rep_c68594_530*), located at 4D (12773232 bp), associated with PL and showing a negative effect of −3.0 cm compared to the wild-type allele (A). Interestingly, the second highest significant marker (*BS00047691_51*) on 7 A (118326946 bp), associated with PL, showed a positive effect of allele C demonstrating the natural phenotypic and genotypic variation of PL. Another very significant marker (*RAC875_c11899_366*), located at 1 A (58704752 bp), and associated with GN, had a positive effect of five extra grains.

Further associated genomic regions 1B (142834452–278473822 bp), 2 A (61286655–61287719 bp), 2D (650322702–650327159 bp), 3B (205465371–222192694 bp), 4B (561513452–561513452 bp), 4D (54447055–69850689 bp), 5B (678917307–678917517 bp), 6A (605564351–605564351 bp), and 7B (450280079–451655622 bp), showed high and consistent effects of SNPs on agronomic traits (Fig. 3b and Supplementary Table 3). For instance, the 1B region was associated with FSS, StS, and TKW by 14 SNPs, 2A with HD and FL, 2D with HI, whereas the 5B genomic region was associated with GY. To test their impact on the associated traits, and to define the most important SNP(s) and trait(s) further analyses were done.

The 118 SNPs passing FDR at 0.01 have been used in the genotype-phenotype network of multi-SNPs with multi-traits analysis. The linkage map between SNPs showed that 24 SNPs are having intra-chromosomal interactions and are presented in 10 chromosomal groups (Supplementary Fig. 3). The analysis showed that the phenotype of agronomic traits is mostly controlled by 24 SNPs (Fig. 3c). The rest of the SNPs did not show connections with the agronomic traits or intra- and inter-chromosomal interactions, therefore, they were excluded from the further analysis (Supplementary Fig 3). The network reveals that 14 SNPs from 1B, 2A, 2D, and 5B (Supplementary Table 4) directly interact with many of the agronomic traits (Fig. 3c). For instance, eight markers from 1A are connected with TKW, StS, FL, HD, HI and FSS, in addition to their interactions with other SNPs (e.g., SNP17 at 5B, which is also connected with GY). The 14 SNPs at 1B, 2A, 2D, and 5B internally interacted (intrachromosomal interactions), and grouped based on their genotypic information and LD (Fig. 3c).

Our findings regarding phenotypic traits could define the key agronomic traits that are connected directly with the most important SNPs and other traits. For example, FSS has a connection with GNFS, StS, HD, GW, and GY. The network showed the most important SNPs controlling many agronomic traits directly or through interaction with other SNPs. For example, SNP2 is interacting with SNP1, SNP3, SNP4, and SNP17, which have further intra- and inter-chromosomal interactions with other SNPs and traits (Fig. 3c). Finally, SNPs within the most significant regions at 1B, 2A, 2D, and 5B (Supplementary Table 4) were used to define the candidate genes.

### Candidate genes linked to agronomic traits

**Figure 4:**
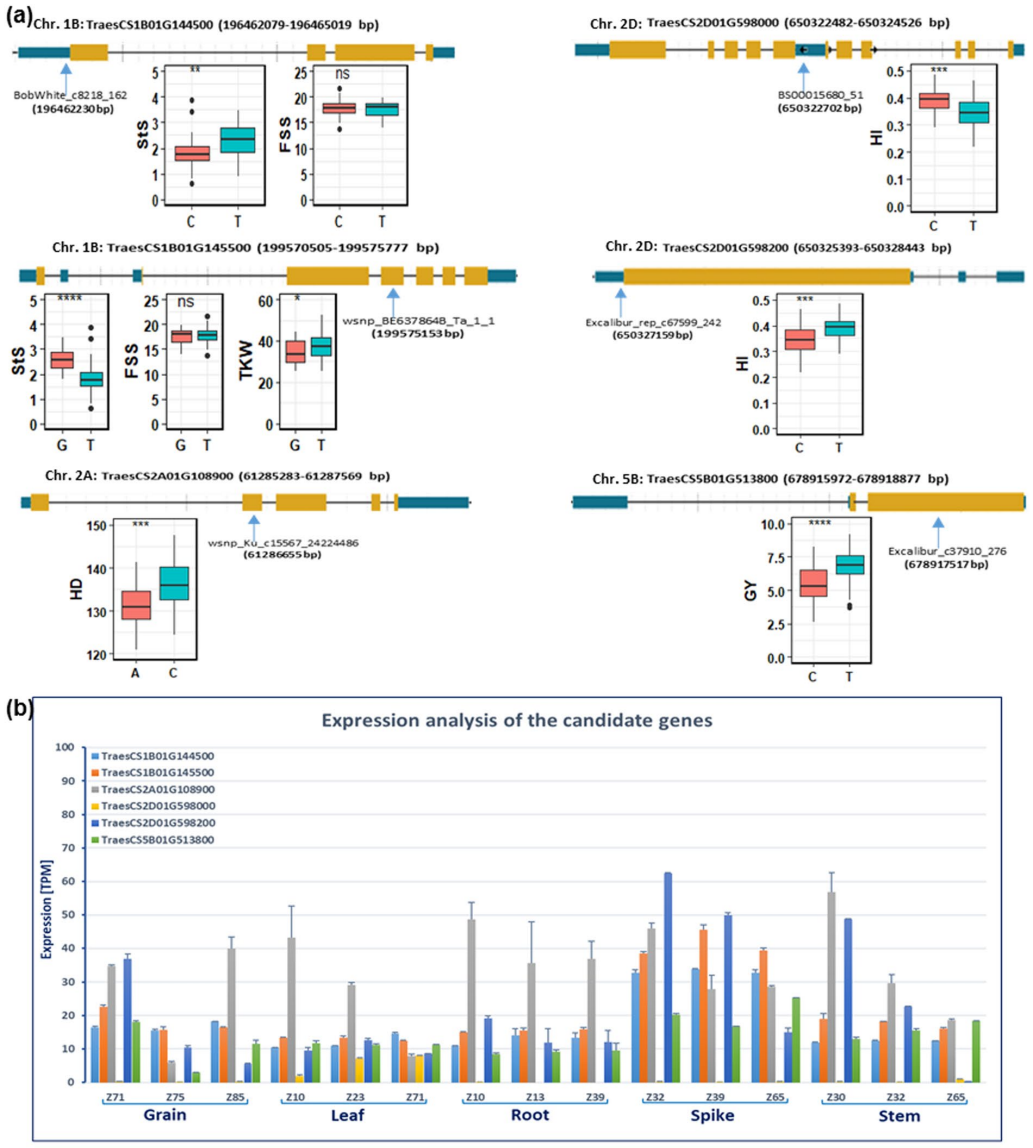
The structure of the candidate genes with the position of the co-located SNP within the gene and SNP-gene haplotype analysis (**a**). The degree of significance indicated as *P, 0.05; **P, 0.01; ***P, 0.001; ns: not significant. Expression analysis of the candidate genes in different wheat plant organs at different developmental stages according to Zadoks, *et al*.^56^ (**b**). GY – grain yield, StS – sterile spikelets per spike, FSS - fertile spikelet per spike, TKW - thousand kernel weight, HD – heading date and HI – harvest index.

Fourteen SNPs at 1B, 2A, 2D, and 5B, found to be highly associated with traits, were used to identify putative candidate genes (Supplementary Table 4). Six SNPs are colocated within putative candidates (Fig. 4a and Table 1). For example, at 1B, markers *BobWhite_c8218_162* and *wsnp_BE637864B_Ta_1_1* were detected at Coding Sequence 1 (CDS1) and Exon4, within the genes *TraesCS1B01G144500* and *TraesCS1B01G145500*, respectively (Fig. 4a and Supplementary Table 4). The allele analysis of these SNPs showed that alleles C and G from these markers have a highly significant impact on StS (Fig. 4a). The gene *TraesCS2A01G108900* at 2A controlled HD via a marker *wsnp_Ku_c15567_24224486* on Exon2, whereas allele A decreased HD significantly (Fig. 4a).

**Table 1:** List of the co-located SNPs within the candidate genes including their associated traits, physical position, and annotation.

SNP #, name & position				Traits	HC gene ID & physical position			SNP_Gene	Description
1	BobWhite_c8218_162	1B	196462230	StS, FSS	TraesCS1B01G144500	196462079	196465019	CDS1	Metal ion transport
2	wsnp_BE637864B_Ta_1_1	1B	199575153	StS, FSS, TKW	TraesCS1B01G145500	199570505	199575777	Exon4	Gibberellin 2-oxidases (GA2oxs)
11	wsnp_Ku_c15567_24224486	2A	61286655	HD	TraesCS2A01G108900	61285283	61287569	Exon2	Ribosomal protein large (RPL19)
13	BS00015680_51	2D	650322702	HI	TraesCS2D01G598000	650322482	650324526	CDS2	Glutathione peroxidase
14	Excalibur_rep_c67599_242	2D	650327159	HI	TraesCS2D01G598200	650325393	650328443	CDS1	C2 calcium/lipid-binding (CLB) and GRAM domain protein
17	Excalibur_c37910_276	5B	678917517	GY	TraesCS5B01G513800	678915972	678918877	Exon2	Bifunctional helicase and thymine dioxygenase JBP2

Two genes (*TraesCS2D01G598000* and *TraesCS2D01G598200*) at 2D were associated with HI by two SNPs located in CDS regions, demonstrating the impact of the allelic variation of these SNPs on HI (Fig. 4a). Finally, GY was controlled by SNP located at Exon2 of the *TraesCS5B01G513800* gene, with the high impact of allele T on final GY (Fig. 4a). Interestingly these genes are involved in enzymes, hormone response, metal ion transport, and response to oxidative stress (Table 1 and Supplementary Table 4) (e.g., genes involved in metal ion transport and gibberellin-controlled spikelet sterility).

### Expression analysis of candidate genes

The expression analysis of candidate genes in different organs, at three developmental stages of each organ, showed a wide range of expression for the genes (Fig. 4b). Generally, gene *TraesCS2A01G108900* at 2A, and gene *TraesCS2D01G598200* at 2D, showed the highest expression in most of the organs at all developmental stages. The expression of gene *TraesCS2A01G108900* was very high during the development of grain, leaf, root, and stem, whereas gene *TraesCS2D01G598200* was highly expressed during spike development (Fig. 4b). Their expression showed that they play biological roles not only with associated traits, but also with other traits during the plant growth and development. The expression of these genes was followed by genes *TraesCS1B01G144500* and *TraesCS1B01G145500*, particularly in grain and spike, demonstrating their roles in spikelet development. Two of the highly associated genes were very low expressed (*TraesCS2D01G598000* and *TraesCS5B01G513800*) in the organs compared with the aforementioned genes (Fig. 4b).

### Genomic prediction of yield-related traits

**Figure 5:**
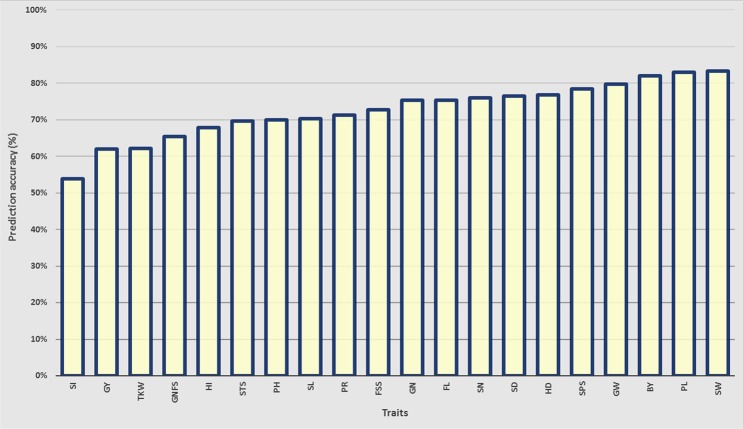
The genome-wide prediction accuracy values obtained by using rrBLUP model for traits: BY - biological yield, GY – grain yield, HD – heading date, HI – harvest index, PL – peduncle length, FL – flowering time, PH – plant height, Pr – protein content, Sd - sedimentation value, SL – spike length, SN - spike number per m^2^, SW – spike weight, SI – spike index, GW – grain weight per spike, TKW - thousand kernel weight, GN - grain number per spike, SPS - spikelets per spike, StS – sterile spikelets per spike, FSS - fertile spikelet per spike and GNFS - grain number per fertile spikelets.

BLUEs for the yield-related traits in the population were used to predict GEBVs using ridge regression best linear unbiased prediction (rrBLUP), which is the most robust model for predicting single yield-related traits. The accuracy of GEBVs ranged from 54% for spike index (SI) to 83% for PL and SW (Fig. 5). GW and biological yield (BY) also have high prediction accuracy values of more than 80%, while most of the studied traits ranged between 62–78% (Fig. 5), supporting the potential of applying GP to enhance genetic gain for such yield-related complex traits hexaploid wheat.

## Discussion

The wheat collection used in the current study is considered as a source for genetic studies in breeding programs across the world, since it includes a founder genotypes^7,21^. High natural phenotypic variation in the wheat collection has been demonstrated for agronomic and developmental traits, as well as drought adaptive traits^7,11,22,23^. In the current study, the analysis of the phenotypic variation using the estimated means (BLUEs) was more informative in the context of natural variation compared with previous studies^11^. The association results showed that SNP array density (15 K) and GWAS analysis strategy were strong enough to identify highly significant marker-trait associations, compared with previous associations identified using DArT^11,23^ and SNP^7,22^ markers. The power of GWAS can be attributed to the utilization of high-density SNPs, and a statistical model that includes PCA and a kinship matrix for controlling the population in addition to the validation of the significant associations.

Decreasing costs of genotyping using high-density SNP-arrays, and development of statistical methods to accurately predict marker effects, have led to the breakthrough in GP. Most previous studies reported that selection decisions based on genome-wide prediction results could improve the accuracy of selection and speed of genetic improvement^13^, and in the most cases, prediction accuracies are sufficient to make GP more efficient than phenotypic selection. The obtained moderate to high accuracies of predication for various traits in this study support the feasibility of applying genomic prediction as a cost-effective means to enhance genetic gain. In particular for traits like spikelet fertility, which is important for wheat grain yield improvment but difficult to determine in a large population. Breeders can utilize GEBVs for genotypes to make selction of parents with high breeding value for the purpose of designing crosses in the breeding program. Such approach leds to speed-up breeding and accelerate the rate of genetic gain of high spikelet fertilty that in turn boosting wheat grain yield.

In the current study, we used the recently developed statistical and bioinformatics analyses, that so-called “genotype-phenotype network”, for multi-loci and multi-traits^20^, which had never been used in wheat. Such analysis empowered us to define the most important SNP(s) underlying complex traits. The newly added spikelet related-traits in this study demonstrated the importance of studying such traits which showed high natural variation. The natural variation of spikelet fertility/sterility traits and grain-yield-related traits has been recently explored in wheat^8,24^.

It has been shown that a negative correlation between TKW with SPS and GN in Chinese wheat^24^ might be a consequence of trade-offs between GN and TKW with BY and HI. Such negative correlation restricts the grain yield and genetic gains of these traits^25^, or a competition for assimilates between spikelets that leads to unbalanced distribution of GN within FSS and florets along the spike^8^. The positive relationships between FSS (or FSS%), GNFS with BY, TKW, HI, SPS and GN that found in the current study, indicate that improving grain yield through enhancing spikelet fertility together with TKW, SPS and BY is plausible. It might be attributed to the genetic material (ancient cultivars) used in the current study which are carrying allele(s) that not present, or present at low frequency in breeding lines or elite/modern cultivars used in the previous studies^8,24,25^. The elite/modern cultivars were selected for a specific purpose based on origin^8^, and always characterized with low number of kernels per spikelet, and less resistance/tolerance to biotic/abiotic stresses compared with old cultivars^26^. Even though this panel was not chosen to study such traits (i.e. spikelet fertility/sterility), it shows high natural variation in these traits, and confirms that the population presents a good donor for improving such traits. The findings are also important in the identification of associated alleles/loci with these traits that could be a valuable source for wheat improvement.

Based on the statistical and bioinformatics analyses done post GWAS, that is “SNP-phenotype network of multi-loci/SNPs and multi-traits” and “SNP-Gene based haplotype analysis”, we were able to discover candidate genes^56^. The structure and function of these candidate genes were analyzed (Fig. 4a). In the present study, six putative genes having SNPs within their physical positions were identified which are associated with agronomic traits, especially spikelet fertility and sterility (Fig. 4a). The gene annotations show their involvement in spikelet sterility and grain yield (Table 1)

We found that *TraesCS1B01G144500* gene is annotated as metal ion transport, and associated with both StS and FSS. The allelic diversity analysis shows that this gene has a significant impact on StS. It was previously shown that metal ion transporters are involved in the uptake of mainly heavy metals in the roots, to be transferred to the shoots via the xylem-phloem transport system, and finally to the grains^27^. The gene is also involved in leaf and inflorescence development in barley^28^. There was a report about 55 metal ion transporter genes, of which 18 were involved in zinc(Zn)/iron(Fe) transport, 13 for copper (Cu), and 10 belong to the Natural Resistance-Associated Macrophage Protein (NRAMP) family^29^. Several transporters belonging to different protein families (e.g., ZIP, NRAMP, Copper Transporter family (Ctr) and NAC) have been shown to regulate growth and development. Cu has a large role in the basic metabolic processes, especially photosynthesis and respiration, while Cu deficiency showed a high impact on plant growth^30^. Cu had a strong effect on reproductive growth and development in cereals by reducing pollen viability and increasing spikelet sterility in wheat^31^. Therefore, it is plausible to suggest that the Cu deficiency in the spike and/or spikelet may result in increasing spikelet and floret sterility/abortion. Therefore, understanding the mechanisms that may optimize Cu in the spike/spikelet is needed.

The *NAM* (No Apical Merstimes) that encodes a transcription factor of the NAC family, is known to be involved in the spike architecture and spikelet sterility^32^, in addition, to enhance salt and drought tolerance^33^. The *NAM-B1* allele, which is solely present in wild emmer wheat, accelerates senescence and increases nutrient remobilization from leaves to developing grain. while the modern wheat cultivars carry the nonfunctional allele^34^. The expression analysis of *NAM* alleles showed high expression of *NAM-A1, -B1* and *-D1* in the stamens and spikelets, while *NAM-B1* is also expressed in the flag leaf^35^. This can explain the spikelet/floret sterility in the modern cultivars that are carrying alleles *NAM-A1* and *NAM-D1*, and demonstrates the role of *NAM-B1* allele in spikelet development in our collection, because of improving nitrogen use efficiency and grain yield.

The allelic diversity analysis of SNPs located in exon 4 of the gene *TraesCS1B01G145500* that are annotated as gibberellin 2-oxidases (GA2oxs) shows a strong impact on the spikelet sterility and TKW (Table 1 and Fig. 4a). The pivotal role of GA2oxs in controlling growth and development (e.g., tillering, plant height, leaf development) are known in cereal crops^36–38^, but their role in spikelet sterility has not been elucidated. Previous studies showed that GA is important for spikelet fertility in crops^39–41^, while the exogenous GA treatment enhances spike development under short-day conditions^42^, and fertile florets number but not grain set if GA is applied at terminal spikelet stage^43^. The spikelet and floret abortion are most likely attributed to GA deficiency at the tip of the spike and spikelet^40^. The expression analysis of this gene (Fig. 4b) during the spike and spikelet developmental stages started from terminal spikelet (Z32) to anthesis at Z65, demonstrating the influence of endogenous GA2oxs on the spikelet/floret sterility. Taken together, we provide evidence that the GA2oxs has an impact on the spikelet/floret sterility in wheat.

Highly significant expression of the *TraesCS2A01G108900* gene that belongs to ribosomal protein large (RPL19) in many wheat developmental organs at different developmental stages suggests its role in wheat growth and development. RPL19 was highly expressed in the shoot and root of rice under drought stress, suggesting its responsibility for drought tolerance and improvement of water use efficiency^44^. In accordance with our results, the gene was associated with HD and expressed in grain and spike, indicating that RPL19 is involved in the stress-tolerance pathway through earliness of the development, which might have influenced the grain yield.

The allelic variation at the locus of the gene *TraesCS2D01G598200* that annotated as C2 calcium/lipid-binding (CLB), and Glucosyltransferases, Rab-like GTPase activators and Myotubularin (GRAM) domain protein, showed its influence on HI. The gene was highly expressed in grain, spike, and stem, indicating that the gene is involved in grain and biomass production which are the main components of HI (Fig. 4a,b). The loss of the *CLB* gene function in *Arabidopsis thaliana* enhanced abiotic stress tolerance (e.g., drought and salt)^45^. The gene was highly expressed in the leaf, root, stem, and flower of *Arabidopsis*. Here we found the same trend of expression in our collection, suggesting the role of this gene in abiotic stress tolerance in wheat. It had been shown that the gene encodes a C2-GRAM domain-containing protein, *Oryza sativa no pollen* (*Osnop*) gene plays a critical role in male gametophyte development during late development of pollen and its germination that might affect grain yield in rice^46^. The pivotal role of C2-CLB and -GRAM families genes might emanate from its crucial function in controlling many complex traits (e.g., grain and biomass) that need to be discovered in wheat.

In conclusion, we showed the value of using such wheat germplasm as donors in breeding for improving grain yield through enhancing TKW, SPS and BY into elite wheat cultivars. High genomic prediction accuracy for the studied traits suggest their usefulness in breeding programs. Particularly, medium to high genomic prediction accuracies (>70%) for spike fertility-related traits (SW, FSS, and SPS) suggests that GP can be implemented to predict breeding values, and facilitate rapid gains from a selection of these traits, in order to boost yield. Applying the GWAS and network analyses presented here enabled us to uncover the key SNPs and yield traits in wheat. The identified SNPs associated with the traits (e.g., FSF and StS) could be valuable for wheat improvement. Moreover, the bioinformatics analysis to detect the candidate genes controlling complex traits provide a solid starting point for functional studies. Our findings here empowered us to tentatively suggest the effective role of these genes in wheat grain improvement through controlling the spikelet sterility. SNP-Gene based haplotype and expression analyses of the candidate genes demonstrate their role in many biological processes, including spikelet development.

## Materials and Methods

### Germplasm and phenotyping

A set of 710 wheat accessions was selected from 2,500 accessions (Novi Sad Core Collection, Institute of Field and Vegetable Crops, Serbia) and phenotyped for more than 54 traits^18^. Based on their phenotypic and genetic variation, a subset collection of 96 hexaploid wheat accessions had been selected and analyzed in the current study. The selected accessions showed high diversity in the 26 developmental, quality, yield and yield-related traits and are originally from 21 countries across five continents^18^. More information regarding the accessions of the population including the method of evaluation and selection is available in Kobiljski, *et al*.^18^. The population was grown under field conditions for up to eight growing seasons (1993–2001) at Novi Sad, Serbia, to phenotype 18 most important agronomic traits, from three plots per season of each accession. Based on these data, four highly spikelet fertility-related traits were calculated. FSS was calculated as the differences between the total SPS and StS. The division of total GN by FSS gives GNFS. FSS and StS were also presented as a percentage, to show their contribution to the SPS. *H*^2^ for each trait over environments (years) was calculated using the following equation:

$${H}^{2}=\frac{{\sigma }^{2}G}{{\sigma }^{2}G+(\frac{{\sigma }^{2}G\times E}{E})+(\frac{{\sigma }^{2}e}{Er})}$$

where *σ*^*2*^*G* is the variance of the genotype, σ^2^E is the variance component of the environments, σ^2^G × E is the variance component of the interaction G × E, *σ*^*2*^*e* represents the variance of the error, and *E* is the number of the environments, and r is the number of replicates.

Analysis of variance was calculated to check whether there were a significant effect of genotypes and/or environments, considering G × E interaction. BLUEs value of each accession for each trait had been calculated, applying residual maximum likelihood (REML) in MLMs to estimate phenotypic means. The accessions were considered as a fixed factor, whereas year was treated as a random factor, and the G × E interaction was incorporated into the analysis. All of these phenotypic analyses and calculations were implemented using GenStat v18^47^. Correlation matrix analysis between the traits and boxplots of each trait were calculated using PAST software^48^.

### Genotyping and population structure

The wheat core collection was genotyped using a 15 k Infinium SNP array that was developed by TraitGenetics GmbH (http://www.traitgenetics.com). The 15 k SNP-array is an improved version of the 90 k iSELECT SNP- array described by^49^. For our GWAS analyses, we used 10,653 SNPs, which had genetic and physical positions based on IWGSC RefSeq v1.0^12^. SNPs with MAF ≥ 5%, and passing quality checking, filtering, and evaluating criteria were used for association analyses. The population structure was determined using principal component analysis (PCA) for obtaining clusters of the accessions, based on their country and/or origin of the region^50^. A kinship matrix was also calculated using the EMMA algorithm within GAPIT^51^, to show the clusters among accessions and familial relatedness based on the polymorphic markers.

### Genome-wide analyses

The association analysis between individual markers of the 10,653 SNPs with the BLUE value of each accession for each trait was performed using the GAPIT R package^51^. Three statistical models were tested, GLM, MLM, and CMLM, to compare their strength and power of association detection. The (QQ) plot was used for assessing how strong the used model in GWAS. FDR was calculated for each trait at the significance level of 0.01 separately. Association signals that passed the threshold of FDR at 0.01 (-log10 P-values ≥ FDR) were considered as significant SNPs and used in further analyses as recommended by^14^

The genome-wide prediction was made using rrBLUP. The model was fitted in R (R Development Core Team, 2016), using rrBLUP package, v4.4^52^. GEBVs were predicted by first estimating the effects of allele substitutions at the marker loci with statistical models that used phenotypic and genotypic information from the training population (TP). GEBVs for each entry of the validation set (VP) were then estimated by summing up the effects according to the individual’s genotypic makeup. To validate the prediction accuracy, a training population (TP, 75% of the population) was created to estimate the GEBVs for the remaining 25% of the whole population (validation population [VP]) based on the marker effects of the used SNPs. The division of the population into subsets (TP vs. VP) and the selection of accessions in each subset were randomly done, according to the PCA numbers that determine the population structure. Five-hundred cycles of iterations, with the function mixed.solve to optimize the parameter scales in marker-based GP considering the REML method, were used to estimate the variance components^52^. Prediction accuracy was computed as Pearson’s correlation between the predicted values and BLUEs of lines in the validation set within each fold.

### SNP-Gene based haplotype analysis and candidate genes

To better understand the genetic basis, and to detect the most important loci/SNPs (passing the FDR at 0.01) that are directly responsible for the studied traits, the genotype-phenotype network of multi-loci/SNPs and multi-traits were calculated using Network-Based Genome-Wide Association Studies (NETGWAS) R package^20^. The genotype-phenotype network is a complex network analysis that takes into account the genetic data of markers (alleles), phenotypes of agronomic traits, and the interaction among each genetic markers and with traits. The intra- and inter-chromosomal interactions network was also calculated. The most significant SNPs that were directly connected with the agronomic traits were used to identify the high-confidence (HC) putative candidate genes, based on their physical positions using the recently published wheat genome sequence^12^. WheatMine web-based platform, which contains gene annotations, gene models, and the transposable elements, was used to identify the gene ontologies (GO) and InterPro number and description for the potential candidate genes based on IWGS v1.0 and v1.1 (https://urgi.versailles.inra.fr/WheatMine/begin.do).

Only HC candidate genes that have the associated SNPs were used for further validation, applying SNP-Gene based haplotype analyses and expression analyses approaches. Such analyses allowed us to reveal the impact of alleles on the associated traits. The significant differences test between alleles was calculated using *t-*test statistics, to show the impact of each allele on the associated trait(s)^56^. Expression analysis has been done through the WheatExp; an expression database for polyploid wheat (https://wheat.pw.usda.gov/WheatExp/#) that include the RNA-seq expression database for wheat grain layers^53^; wheat grain at three different stages of development^54^; wheat spike, root, leaf, grain and stem organs at different developmental stages^55^; and other databases. RNA-seq are presented as TPM (Transcripts Per Kilobase Million).

## Supplementary information

Supplemental information.
