# Supplementary figures and images for: Genome-wide and SNP network analyses reveal genetic control of spikelet sterility and yield-related traits in wheat
Source: Sci Rep. 2020 Feb 7;10:2098. doi: 10.1038/s41598-020-59004-4 (PMC7005900; doi:10.1038/s41598-020-59004-4)

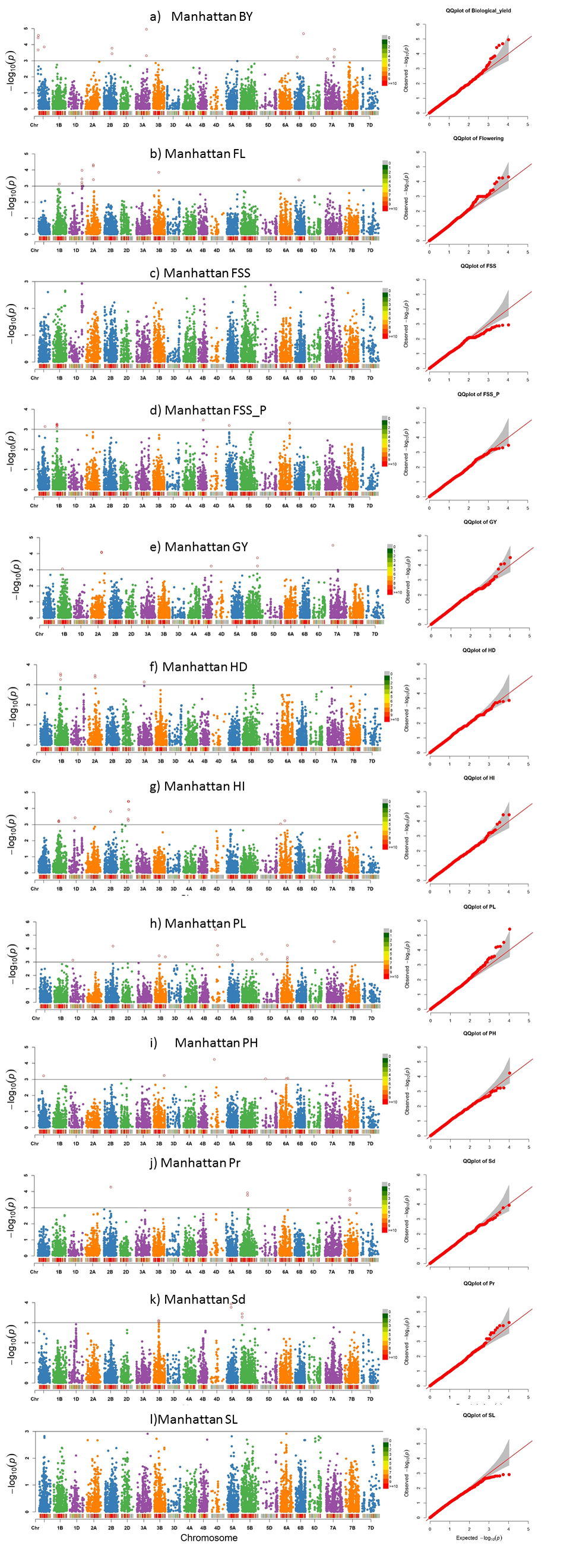

Supplement: Supplementary file 1 — Supplemental information. [file 41598_2020_59004_MOESM1_ESM.zip › FigureS1_300dpi.tif]

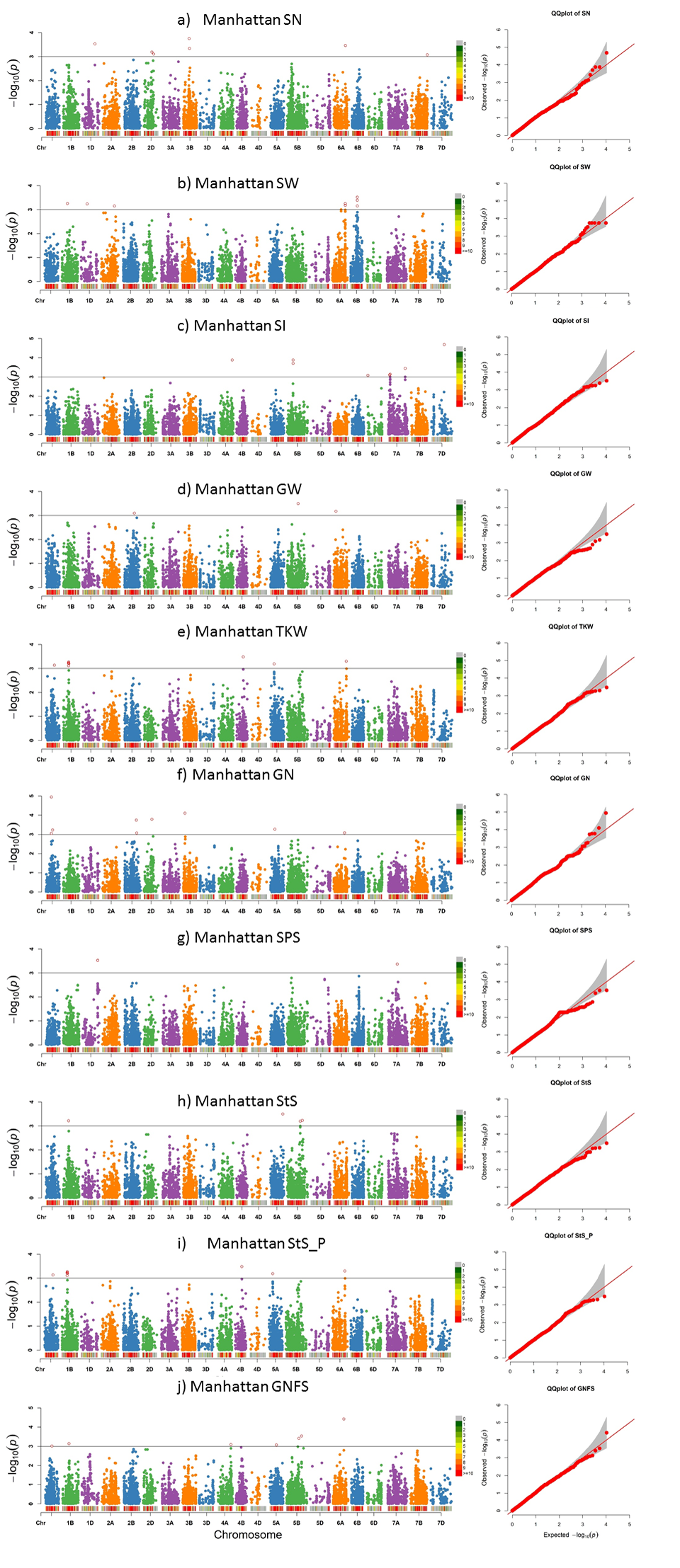

Supplement: Supplementary file 1 — Supplemental information. [file 41598_2020_59004_MOESM1_ESM.zip › FigureS2_300dpi.tif]

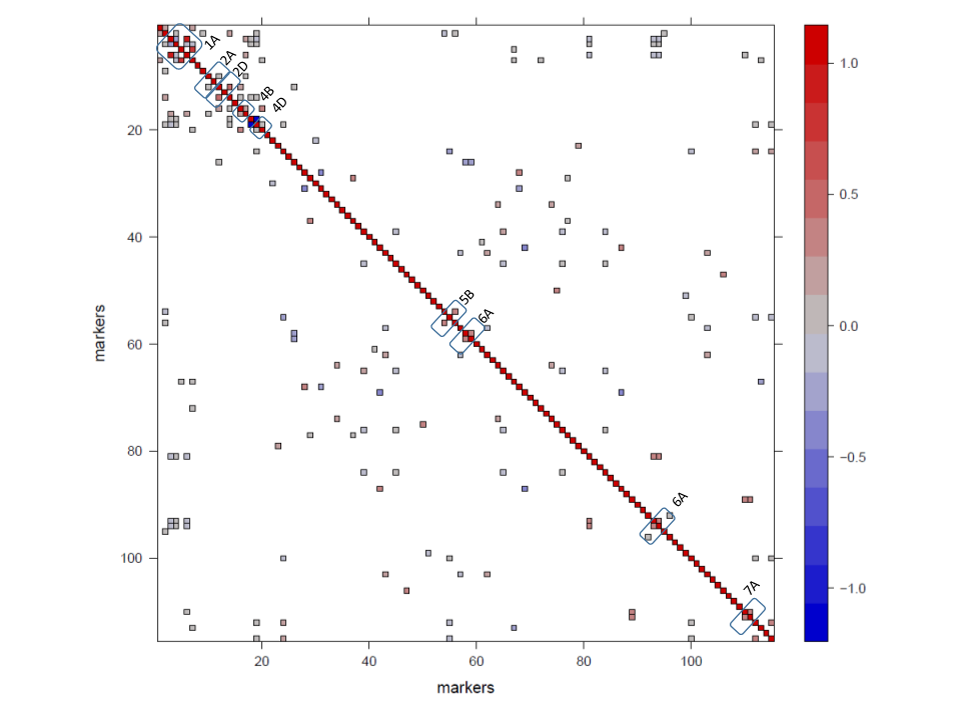

Supplement: Supplementary file 1 — Supplemental information. [file 41598_2020_59004_MOESM1_ESM.zip › FigureS3_300dpi.tif]
